# Synthesis and in situ oxidation of copper micro- and nanoparticles by arc discharge plasma in liquid

**DOI:** 10.1038/s41598-023-41631-2

**Published:** 2023-09-21

**Authors:** Alibek S. Zhakypov, Renata R. Nemkayeva, Yerassyl Yerlanuly, Malika A. Tulegenova, Beibarys Y. Kurbanov, Madi B. Aitzhanov, Aiymkul A. Markhabayeva, Maratbek T. Gabdullin

**Affiliations:** 1 Kazakh-British Technical University, 59 Tole Bi, 050000 Almaty, Kazakhstan; 2 Al-Farabi Kazakh National University, 71 Al-Farabi Av., 050040 Almaty, Kazakhstan; 3 Institute of Applied Science and Information Technologies, Shashkina, 40/48, 050038 Almaty, Kazakhstan

**Keywords:** Chemical physics, Particle physics, Plasma physics

## Abstract

This work presents a one-step controlled method for the synthesis of copper oxide nanoparticles using an arc discharge in deionized water without subsequent thermal annealing. The synthesis conditions were varied by changing the arc discharge current from 2 to 4 A. Scanning electron microscopy images of samples synthesized at discharge current of 2 A revealed the formation of tenorite (CuO) nanopetals with an average length of 550 nm and a width of 100 nm, which had a large surface area. Arc discharge synthesis at 3 and 4 A current modes provides the formation of a combination of CuO nanopetals with spherical cuprite (Cu_2_O) nanoparticles with sizes ranging from 30 to 80 nm. The crystalline phase and elemental composition of the synthesized particles were identified by X-ray diffraction analysis, Raman spectroscopy and Energy dispersive analysis. As the arc discharge current was raised from 2 to 4 A, two notable changes occurred in the synthesized particles: the Cu/O ratio increased, and the particle sizes decreased. At 4 A, the synthesized particles were from 30 to 80 nm in size and had a spherical shape, indicating an increase in the amount of cuprite (Cu_2_O) phase. The optical band gap of the aqueous solutions of copper oxide particles also increased from 2 to 2.34 eV with increasing synthesis current from 2 to 4 A, respectively. This suggests that the proposed synthesis method can be used to tune the band gap of the final material by controlling the Cu/O ratio through the current of arc discharge. Overall, this work demonstrates a novel approach to the synthesis of copper oxide nanoparticles with controllable CuO/Cu_2_O/Cu ratios, which has the potential to be useful in a variety of applications, particularly due to the significant enhancement of photocatalytic abilities and widen the working spectral range.

## Introduction

Metal oxide nanomaterials are becoming increasingly popular due to their physicochemical, electrical and optical properties^1–3^. Based on metal oxides, different semiconductor architectures have been developed, which allows them to be used in various interdisciplinary areas^4^. Copper oxide nanoparticles have shown great potential for applications in medicine^5–7^, agriculture^8–10^, and semiconductor devices^11,12^. One of the most important factors influencing the properties and application of copper oxide nanomaterials is the synthesis of various shapes and structures on their basis. Thus, the most popular synthesized structures are nanowires^13,14^, nanoflowers^15,16^, and spherical nanoparticles^17^, which are used as efficient photocatalysts^18^, gas sensors^19,20^, and supercapacitors^21^. Despite the low toxicity of copper oxide^22^, the synthesis of particles often includes toxic compounds, especially when using a reducing agent in chemical methods^23^. Compared with common methods for the copper oxide nanoparticles synthesis, such as chemical vapor deposition^24^, laser ablation^25^, sol–gel method^26^ and biological synthesis^27^, in-liquid plasma is of particular interest because of the simple working process. The study of in-liquid plasma can demonstrate new interactions between liquid and low-temperature plasma as a new approach to better understand the physical mechanisms of complex plasma systems, the relevance of which is demonstrated in the Plasma Roadmap^28,29^. This method also has the advantage of being a low cost process, since plasma in liquid does not require expensive equipment such as special vacuum chambers and gas lining systems with anti-corrosion materials.

It should be noted that one of the main criteria in the synthesis of copper oxides by an arc discharge besides the electrodes and discharge parameters is the environment around the discharge zone, which can determine the structure, composition and even morphology of the resulting materials. In many studies of electric discharge, water was used as a liquid media due to its unique physicochemical properties and accessibility^30^. For example, in reference^31^, it was demonstrated that by using deionized water as a dielectric medium, spherical CuO nanoparticles were obtained. However, when water was replaced with an argon gas medium, CuO nanowires were formed. In additon, Yang et al. claim the advantage of using pure water medium (chemical-free route) for the green synthesis of copper oxide particles at room temperature through the mechanism of water ionization and subsequent formation of copper hydroxides and then oxides^32^.

One of the common ways to control the phase and composition of the synthesizing copper oxide in gaseous medium usually consists of a variation of the Ar/O2 ratio during arc discharge synthesis. For instance, in ref.^33^ it was shown that increasing oxygen pressure lead to a decrease in the cuprite (Cu_2_O) phase and the prevalent formation of tenorite (CuO). Using this experience it is reasonable to add an additional source of oxygen to in-liquid plasma medium^34^.

It should be noted that copper nanoparticles can also be synthesized using different regimes of discharge. For instance, recent paper of Efimov et al. presents the synthesis of copper oxide nanoparticles using so-called dry aerosol jet printing based on spark discharge process^35^. In^36^, the authors, using a spark discharge in water, managed to control the sizes of the synthesized copper and copper oxide particles by adding HCl in small concentration adjusting the electrical conductivity of water. Considering these techniques one should take into account the energy efficiency of the synthesis process, since spark discharge requires higher voltages as compared to arc discharge process. Moreover, the use of electrolytes can significantly stabilize the arc discharge in a liquid, but they lead to a complication of the reaction, introducing many side electrochemical reactions^30^. Therefore, the proposed technique based on arc discharge in deionazed water is energetically efficient, easy, eco-friendly and fast way to produce copper oxide nanoparticles.

It has been reported that the interaction of the liquid with plasma leads to the formation of reducing agents, which allows to reduce the metal ions faster^37^. In particular, when using an arc discharge in a liquid, the advantage of use of small currents of several amperes^38,39^ has been reported, since the arc discharge generates a high temperature, which can lead to erosion of metal electrodes. Changes in the current during an arc discharge process can lead to the predominance of various processes in the liquid itself, specifically different reactions of ionization and splitting of water molecules^40,41^, which can correspondingly affect the composition and structure of the synthesized particles. Recently, studies have been carried out on obtaining particles from electrodes directly without the use of electrolytes and reducing agents using plasma in water^42^. A discharge in a liquid medium with oxygen content makes it possible to oxidize metal particles without using thermal annealing after synthesis, which makes it possible to control the synthesis of monodisperse nanoparticles of oxides not only of copper, but presumably of many other metals.

Among the variety of metal nanomaterials, Cu-based nanostructures have shown great promise for industrial and environmental applications. However, the poor photocatalytic performance of single-phase Cu nanomaterials limits their effectiveness. To address this issue, the development of a junction combining two or three phases with different bandgaps has been proposed. Such junctions would significantly enhance photocatalytic abilities by promoting efficient charge separation and extending the photoexcitation range. Recent research has shown that the CuO and Cu_2_O phases of oxidized Cu nanoparticles exhibit improved photocatalytic activity under visible light. Therefore, the construction of Cu/CuO/Cu_2_O heterojunctions is an attractive approach for a wide range of environmental applications^43^.

In this study, arc discharge plasma between water-immersed copper electrodes was used as promising and simple process for producing copper oxide particles, where water acted as an oxygen source as well as a dielectric medium. The process is environmentally friendly as no aggressive gases or chemical additives such as electrolytes and reducing agents are used. To obtain an arc discharge in water, a power source with capacity of 1 to 70 μF was used. The arc discharge current was the main parameter that changed the conditions of particle synthesis. In this case, the arc discharge current varied from 2 to 4 Amperes. Copper oxide particles were synthesized from the electrode directly using an arc discharge in water. The workflow was technologically simplified, since the preparation of particles was carried out in one synthesis reactor, without any additives and subsequent thermal treatment. This method made it possible to synthesize copper oxide particles using relatively low arc discharge currents in water without subsequent annealing and to control the oxidation of copper particles during (in situ) synthesis.

## Experimental part

**Figure 1:**
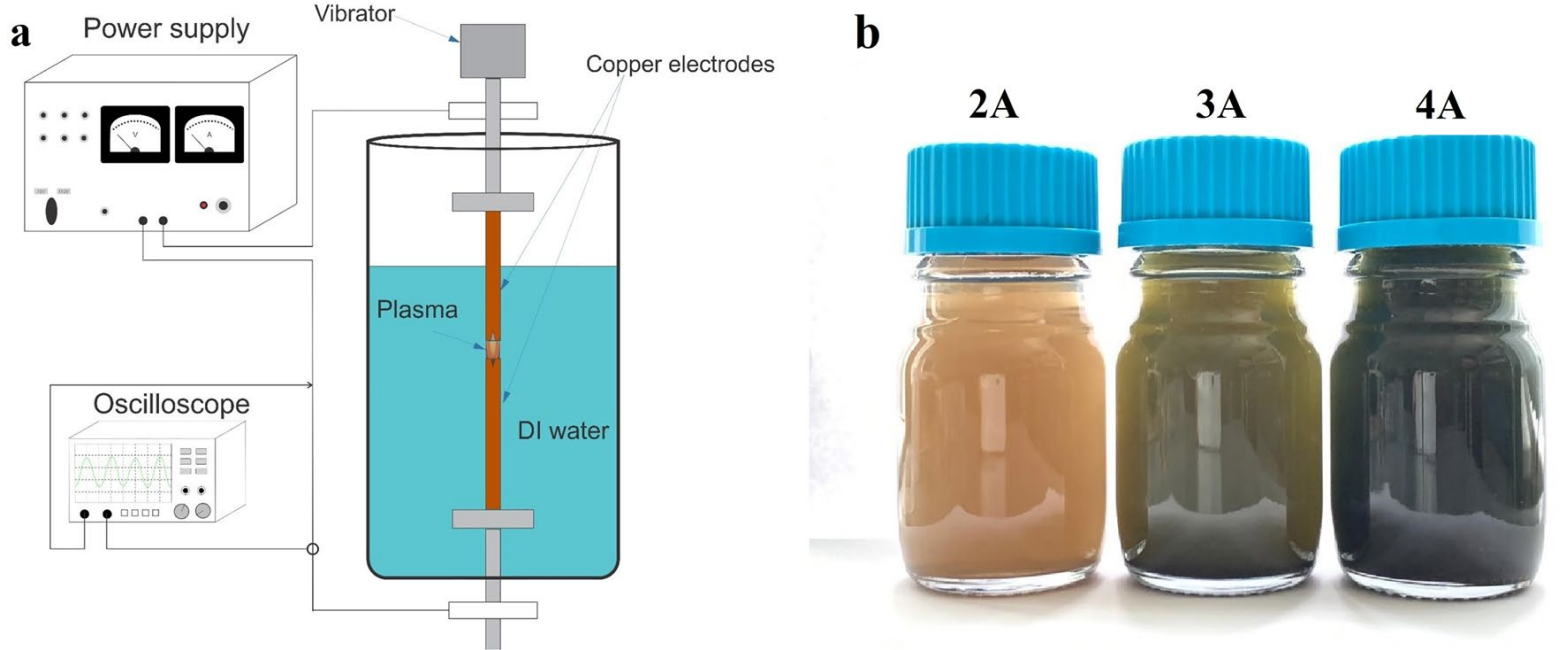
Scheme of the experimental setup (**a**) and colloidal solutions of copper oxide particles obtained at different currents (**b**).

An arc discharge system in deionized water was used to obtain copper oxide particles^44^. Deionized water with a resistivity of 18.2 MΩ*cm was obtained using a Sartorius arium 611DI instrument. The diagram of the arc discharge system is shown in Fig. 1a. The setup consists of an arc discharge source (DC), two copper electrodes 6 mm in diameter, and an oscilloscope for monitoring the current–voltage characteristic. To minimize contamination, such as an oxidized layer, before each experiment, the surface of the electrodes was ground with sandpaper, followed by rinsing with acetone and ethanol. The electrodes were arranged vertically for more convenient control of the gap during the discharge. The arc discharge was created between two copper rods of electrodes completely immersed in deionized water.

The plasma of a pulsed arc discharge in deionized water is created by the energy of capacitors located in the power source. The varied technological parameter of synthesis was the arc discharge current. With the same configuration of copper electrodes, currents of 2 A (1 μF, 100 V), 3 A (30 μF, 50 V) and 4 A (70 μF, 30 V) were passed. The voltage was applied until the breakdown current occurred with separated electrodes. To start the discharge, the electrodes were placed in direct contact without a gap between them. When the electrodes were torn off, a breakdown discharge occurred. After the discharge, the distance between the electrodes was controlled. The working cycle of particles synthesis was 10 min. An electromagnetic vibrator (50 Hz) was used to initiate the cyclic movement of the electrodes. The rate of powder formation during synthesis is 12 mg/min at 2 A, 83 mg/min at 3 A, 97 mg/min at 4 A. As a result of the experiment, a colloidal solution was formed (see Fig. 1b). The study used both a colloidal solution and copper oxide powder extracted from the solution. Pure powder was extracted from the colloidal solution by centrifugal precipitation, then the precipitate was filtered and dried at 120 °C for 1 h.

### Characterization

The elemental composition and morphology of the obtained particles were studied by energy dispersive analysis (EDX) and SEM on a Quanta 200i 3D complex. Structural properties were studied using Raman spectroscopy using a Solver Spectrum spectrometer (NT-MDT) in 180° reflection mode. The excitation source was a laser with a wavelength λ = 473 nm. The diameter of the laser spot on the sample was ~ 2 μm. To carry out studies by scanning electron microscopy and Raman spectroscopy, samples were deposited from a colloidal solution onto the surface of a c-Si film and glass, respectively. The crystalline phases of particles samples were studied using X-ray diffraction analysis on a Rigaku MiniFlex 600 X-ray spectrometer with copper radiation (CuKα). The recording modes are as follows: X-ray tube voltage 40 kV, tube current 15 mA, goniometer movement step 2θ = 0.02°. Phase analysis was performed using the PCPDFWIN program with the PDF-2 diffraction database. To determine the optical properties of the colloidal solution, the optical transmission spectra were studied using a Shimadzu UV-3600 spectrophotometer.

## Results and discussion

During the synthesis of copper particles by an arc discharge in water, a gradual color change was observed from light brown in low-current mode 2A to dark green in synthesis at 4A. Changes in the color of the colloidal solution at the stages of synthesis at different current modes are shown in Fig. 1b.

**Figure 2:**
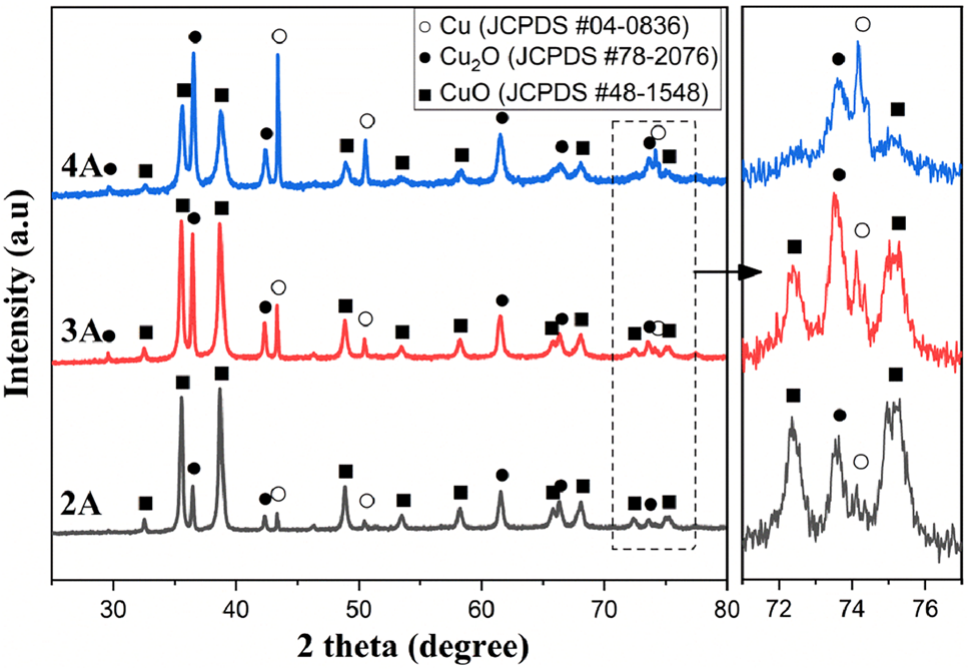
X-Ray diffraction patterns (XRD) of synthesized copper oxide nanoparticles.

The crystal structure and purity of the freshly prepared copper oxide particles were investigated by X-ray diffraction analysis (Cu-Kα radiation). Figure 2 shows XRD patterns of copper oxide nanoparticles obtained by an arc discharge in deionized water at three current modes. In mode 2A, characteristic low-intensity diffraction peaks of copper located at 43.7°, 50.7°, and 74.3° (JCPDS 04-0836) were observed. They correspond to the (111), (200), and (220) planes of the fcc structure, respectively^45^. As the current increases to 3A and 4A, the intensity of the metallic copper peaks increases and a characteristic peak at 74.3° for pure copper phase becomes more prominent^45^. In the case of mode 2A, the peaks characteristic of Cu_2_O at 36.37°, 42.26°, 61.36°, 73.664° and 77.38° correspond to the planes (111), (200), (220), (311) and (222) of the cubic cuprite phase, respectively, and are in good agreement with JCPDS 78-2076^46^. As the current regime increases, a peak appears at 29.51° corresponding to the (110) plane, which also belongs to the Cu_2_O phase. The most intense phase in the low current mode (2A) is the CuO tenorite, which decreases in intensity with an increase in the arc discharge current. Crystallographic orientations arose at 2θ values of 32.6°, 35.5°, 38.6°, 48.8°, 53.5°, 58.2°, 65.9°, 67.82°, 72.17°, and 75.1°, respectively, which corresponds to the (110), (111), (111), (202), (020), (202) (113), (202), (311), and (004) planes of the CuO monoclinic tenorite phase structure, and agrees well with the previously published data (according to JCPDS card 48-1548)^47^.

To evaluate the crystallite size of our samples when changing the current regime, the Scherrer equation was used^46^:

1 $$\mathrm{D}=\frac{\mathrm{K}\cdot \uplambda }{\upbeta \cdot \mathrm{cos\theta }}$$

where D, K, λ, β, θ are the average crystallite size (nm), Scherrer constant, X-ray wavelength, full with at half maximum of the peak, and Bragg angle in degrees, respectively. The results are shown in Table 1. Based on the data, it can be seen that the sizes of crystallites in all structures (tenorite, cuprite and copper) decrease with increasing discharge current.

**Table 1:** Crystallite size of tenorite, cuprite, and metallic copper (CuO, Cu_2_O, and Cu) structures synthesized by arc discharge in deionized water at different current modes.

Current mode	2A			3A			4A		
	CuO	Cu_2_O	Cu	CuO	Cu_2_O	Cu	CuO	Cu_2_O	Cu
Peak position, 2Θ	35.55	36.46	43.31	35.54	36.44	43.31	35.61	36.53	43.39
FWHM	0.27	0.17	0.08	0.3	0.18	0.09	0.41	0.25	0.1
Crystallite size, nm	32.59	52.77	106.54	28.68	48.24	94.22	21.41	34.61	85.64

**Figure 3:**
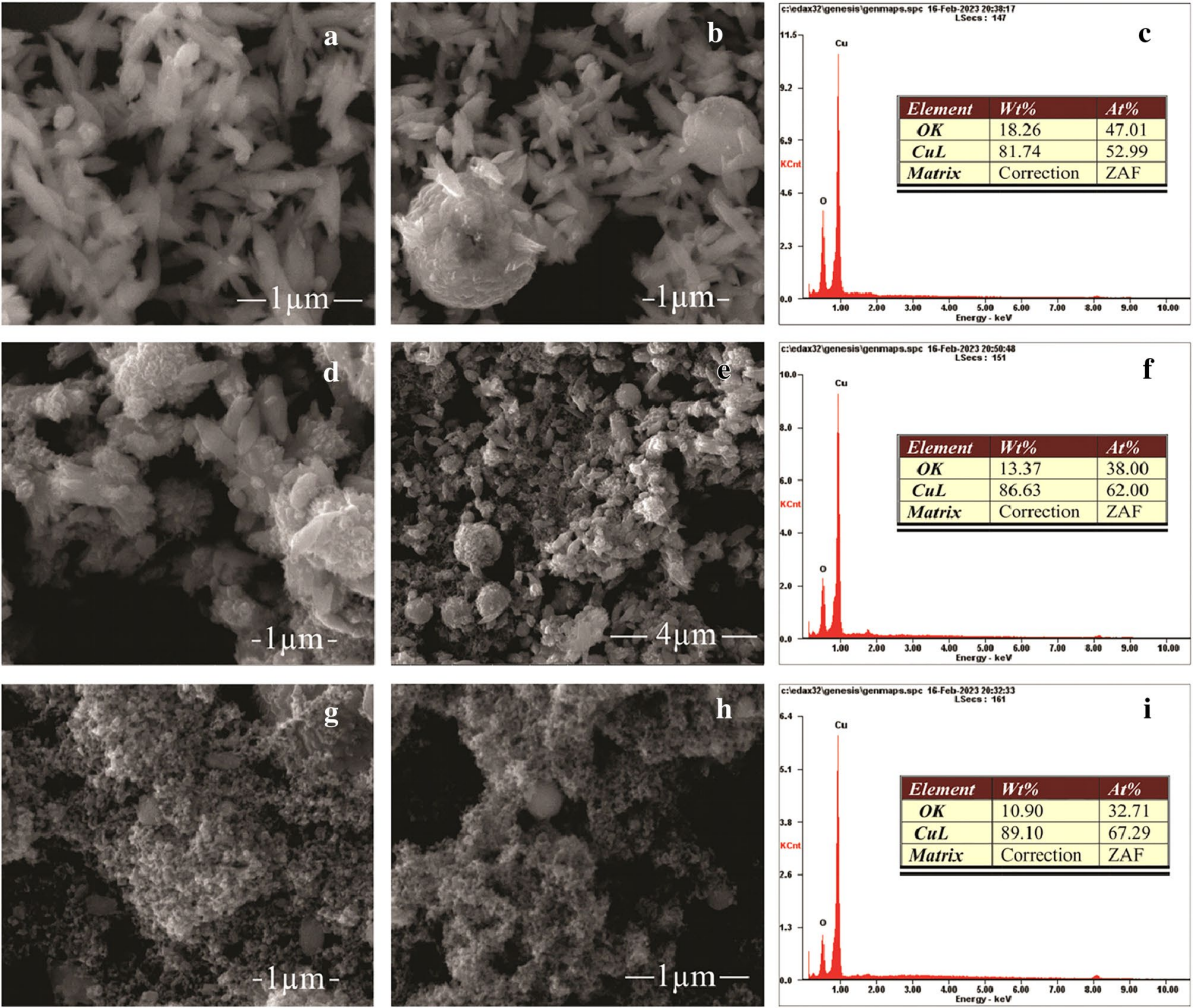
SEM images and Energy-dispersive analysis spectra of copper oxide particles obtained at different current modes by an arc discharge in deionized water: (**a**–**c**)—2A; (**d**–**f**)—3A; (**g**–**i**)—4A.

The morphology of the synthesized copper oxide structures was characterized using a scanning electron microscope (SEM). As shown in Fig. 3a, copper oxide particles formed at 2 A current mode had a petal shape with similar size and morphology. By examining different areas, it was found that the length and width of the particles averaged 550 nm and 170 nm, respectively. In some areas, as can be seen from Fig. 3b, these particles aggregated together forming spherical shapes with sizes reaching 1.7 μm in diameter. Previous research^15,48^ has established that particles with such a morphology can be identified as tenorite (CuO) phase.

With an increase in the current regime to 3 A, the structure retained the shape characteristic of CuO. Figure 3d,e shows SEM images of CuO particles at 3A which exhibit a petal-like morphology with increased agglomeration compared to particles synthesized at the 2A current mode. The length and width of the petals decreased to 400 nm and 110 nm, respectively. The number of spherical particles also increased, spherical particles with a diameter of 800 nm to 1 μm appeared.

Figure 3g–h shows SEM images of copper oxide particles synthesized in 4A mode. The size of monodisperse nanoparticles ranges from 30 to 80 nm. A small amount of non-agglomerated spherical particles up to 120 nm in size can also be observed, presumably having a pure copper phase, which increasing amount at higher currents was previously confirmed by XRD analysis.

The elemental composition of the synthesized particles was determined using energy dispersive analysis (Fig. 3c,f, i). The EDS results confirm that the synthesized particles at a current mode of 2A are pure copper oxide CuO without impurities. Figure 3f shows EDS spectrum of the sample synthesized at 3A, energy dispersive analysis showed a slight increase in copper percentage. Energy dispersive analysis of particles at 4A (Fig. 3i) showed about 32.71 at.% of oxygen and 67.29 at.% of copper, which can approximately indicate the phase of cuprite (Cu_2_O).

**Figure 4:**
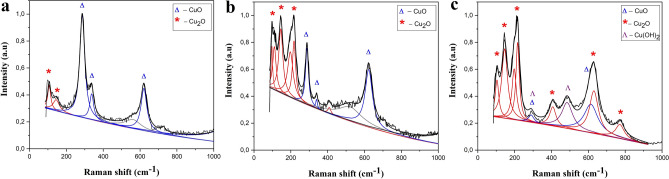
Raman spectra of copper oxide particles synthesized at (**a**) 2A, (**b**) 3A, (**c**) 4A current modes.

To additionally confirm the composition of synthesized particles of the CuO/Cu_2_O complex, the Raman spectra were measured (Fig. 4). As it was shown above, the XRD and EDS data revealed that particles synthesized at 2A current mode mainly correspond to the CuO tenorite phase. The CuO crystal has a monoclinic lattice of space group C2/c. Each copper atom is bonded to four oxygen atoms located at the vertices of an almost rectangular parallelogram^49^. The equation related to lattice vibrations of a primitive cell has the form: ΓRA = (4A_u_ + 5B_u_ + A_g_ + 2B_g_)^50^. As can be seen from the equation, there are 12 zone-central optical phonon modes in the pure CuO phase. Among these 12 modes, there are three acoustic modes (A_u_ + 2B_u_), six infrared active modes (3A_u_ + 3B_g_), and three Raman active modes (A_g_ + 2B_g_)^51^.

Figure 4a shows the spectrum of particles synthesized at 2A current mode, which is characterized by three main peaks at 286, 330 and 620 cm^−1^. The peak at 286 cm^−1^ can be assigned to the A_g_ mode, and the peaks located at 330 cm^−1^ and 620 cm^−1^ can be assigned to the B_1g_ and B_2g_ modes, respectively^52^. According to theoretical calculations, these oscillations correspond to the motion of only oxygen atoms in the CuO lattice, and for the A_g_ mode, the oscillations occur along the b axis, while for the B_g_ mode, the oscillations are perpendicular to the b axis. A slight trend below 200 cm^−1^ can be deconvoluted into two peaks at about 107 and 145 cm^−1^ which are characteristics of Cu_2_O. While the broad peak at ~ 560–570 cm^−1^ can be possibly due to the second order of the A_g_ mode (peak at 286 cm^−1^).

Cu_2_O has a cuprite cubic structure, space group ${\text{Pn}}\overline{3}{\text{m}}$, which consists of an fcc cell of copper atoms and a bcc cell of oxygen atoms. Since there are 6 atoms per unit cell of Cu_2_O, 18 phonon modes are expected according to group theory^53^.

$$ \Gamma = A_{2u} + E_{u} + \, 3T_{1u} + T_{2u} + T_{2g} $$

And only one mode, T_2g_ is Raman-active. A T_2g_ mode is a threefold degenerated mode, which is symmetric with regard to one of the fourfold rotation axis. The "g" shows that there is an inversion center which is kept during the vibration. This mode arises as a result of the motion of two oxygen sublattices relative to each other, while the copper sublattice remains fixed (immobile). However, the appearance of additional Raman peaks can be due to defects in a pure crystal and as a result of overtones and mode combination.

Figure 4b shows the Raman spectrum of particles synthesized at the 3A current mode, where, besides the peaks corresponding to the CuO phase, additional peaks appear at ~ 100, 145, and 218 cm^–1^, indicating the presence of the Cu_2_O phase in the powder composition, which is in good agreement with the XRD and EDS. The three intense peaks two of which are slightly asymmetric can be deconvoluted into 5 bands with positions at 97, 109, 145 cm^−1^, which correspond to T_2u_, E_u_, T_1u_ modes of Cu_2_O, respectively, and 197 and 218 cm^−1^ which are possibly the second order of the peaks at 97 and 109 cm^−1^. Peaks at 410 and 520 cm^−1^ are also due to Cu_2_O vibrations, particularly the band at 410 cm^−1^ is considered as fourth-order overtone, while 520 cm^−1^ corresponds to T_2g_ Raman active mode. Less intensive peaks at 287, 339 cm^−1^ indicate the presence of CuO phase. While rather broad band at about 623 cm^−1^ can be attributed to both CuO (B_g_ mode) and Cu_2_O (T_1u_ mode). The peak at 145 cm^−1^ corresponding to the T_1u_ cuprite mode can appear in the Raman spectrum as a result of oxygen vacancies^54–56^. The low intensity of the Raman peaks of copper oxides may indicate the presence of a significant amount of the metal phase in the composition of the samples, which is also confirmed by the XRD data.

The Raman spectrum of samples obtained at 4A (Fig. 4c) is represented by a large number of peaks, mainly related to the Cu_2_O phase—peaks at 105, 145, 192, 214, 410 and 770 cm^−1^. A low-intensity broad peak at 290 cm^−1^ can be caused by the presence of Cu_2_O and/or Cu(OH)_2_, which is quite understandable, since the synthesis of nanoparticles is carried out in water. The presence of the copper hydroxide phase is also indicated by a peak in the region of 490 cm^−1^. The broad peak in the region of 630 cm^−1^ can be decomposed into two components: at 620 cm^−1^ corresponding to CuO and at 630 cm^−1^ characteristic of the Cu_2_O phase.

Thus, it was again shown by Raman spectroscopy that by changing the current regime of synthesis, it is possible to control the degree of oxidation of the obtained particles with predominant content of CuO in the case of 2A, and Cu_2_O in the case of 3 and 4 A.

**Figure 5:**
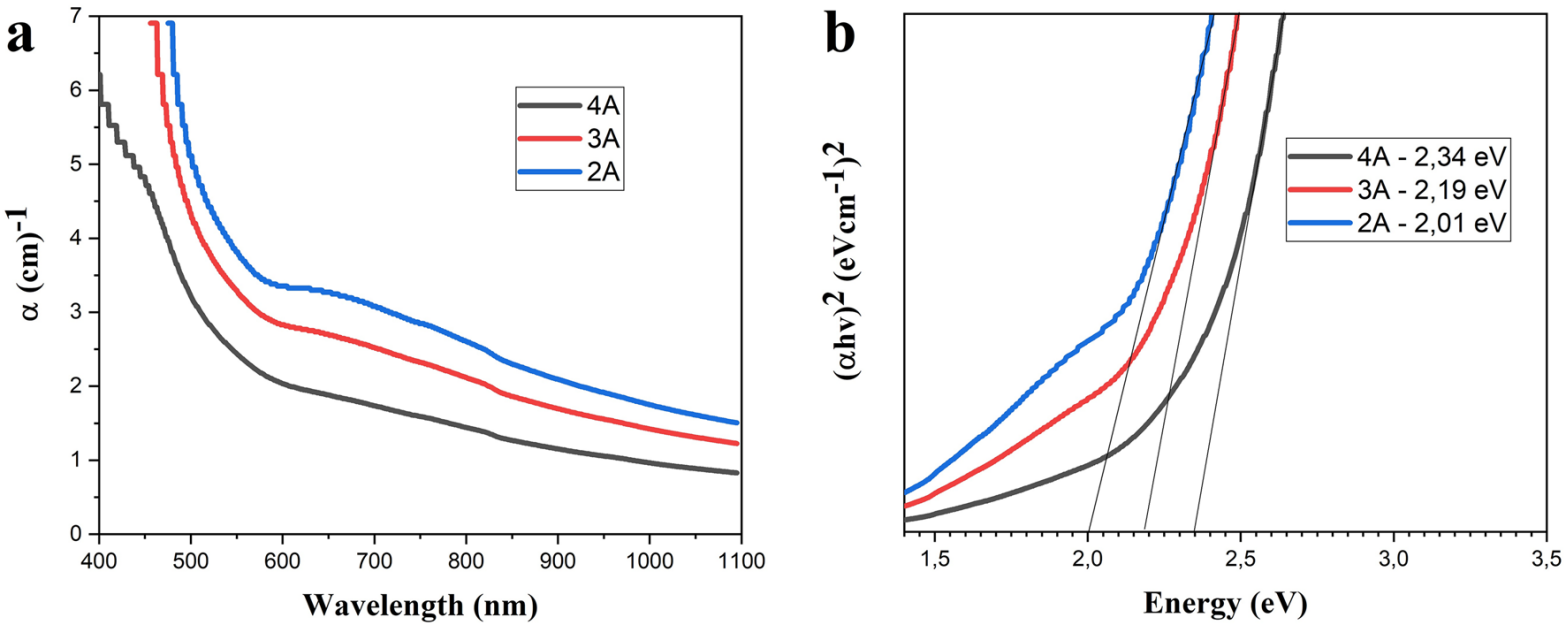
UV–VIS absorption spectrum (**a**) and determination of optical band gap values (**b**) of colloidal solutions obtained at different current modes.

The optical properties of the synthesized colloidal solutions of copper oxide were analyzed using a Shimadzu UV-3600 spectrophotometer in the visible range to calculate the light absorption zone edge and the optical band gap. Figure 5a shows the spectrum of the optical absorption edge of fresh colloidal solutions synthesized at various current modes (2–4 A). The absorption of light in the visible region was most intense for the solutions synthesized at 2A. The solutions synthesized at 3A and 4A also show a wide absorption region, but with a noticeably lower intensity. The optical band gap of copper oxide samples can be calculated using the Tauc equation:

$$\alpha hv=A{(hv-{E}_{g})}^\frac{n}{2}$$

where n—electron transition between conduction band and valence band, in our case used n = 4 for allowed indirect transition^57^. A plot of (αhν)^2^ versus energy (hν) for determining the optical band gap (E_g_) is shown in Fig. 5b. The calculated optical band gap of the colloidal solution at a current mode of 2A was 2 eV. And as the arc discharge current increased, the band gap also gradually increased, reaching 2.34 eV for sample synthesized at 4A.

It is worth noting that the typical band gaps for bulk CuO and Cu_2_O are 1.24 eV and 2.47 eV, respectively^58^. However, that the band gap of copper oxide can be influenced by factors such as morphology and size. According to^57^, the band gap of CuO nanopetal is about 1.73 eV, which is higher than that of bulk CuO, while in ref.^59^ authors claim that a decrease in the size of crystallites can also increase the band gap. And as was observed in our experiment, an increase in current value leads to a decrease in crystallite sizes of all forming phases—CuO, Cu_2_O and Cu. In addition, the presence of the Cu_2_O phase in the synthesized samples, which was revealed by XRD, EDS, and Raman data, can further contribute to increasing the band gap. Thus, it can be assumed that the band gap of the synthesized copper oxides is determined by three factors CuO/Cu_2_O phase content, changes in morphology, and a decrease in crystallite sizes as the current value increases.

## Conclusion

In the work, the one-step method of synthesizing copper oxide nanoparticles using an arc discharge in deionized water without subsequent thermal annealing has been presented. The study varied the synthesis conditions by changing the arc discharge current, resulting in nanoparticles with varying sizes and shapes. Elemental composition as well as Raman and XRD analysis showed the formation of combination of Cu_2_O/CuO/Cu phases with different ratios depending on discharge current mode. The band gap of the aqueous solutions of copper oxide particles increased with increasing synthesis current, indicating the proposed method's ability to tune the band gap of the final material by controlling the Cu/O ratio, morphology and crystallite sizes. The combination of CuO/Cu_2_O nanoparticles offers several advantages, particularly it can improve the efficiency of the photocatalytic processes by providing a synergistic effect between the two types of nanoparticles. This synergistic effect can enhance the photocatalytic properties of the system, such as improved light absorption, charge separation, and redox reactions, resulting in higher efficiency and effectiveness in various applications, including water treatment, air purification, and solar energy conversion.

## Data Availability

The data presented in this study are available on request from the corresponding author.
